# Intramuscular injection of Botox causes tendon atrophy by induction of senescence of tendon-derived stem cells

**DOI:** 10.1186/s13287-020-02084-w

**Published:** 2021-01-07

**Authors:** Peilin Chen, Ziming Chen, Christopher Mitchell, Junjie Gao, Lianzhi Chen, Allan Wang, Toby Leys, Euphemie Landao-Bassonga, Qiujian Zheng, Tao Wang, Minghao Zheng

**Affiliations:** 1 Center for Orthopaedic Translational Research, Medical School, University of Western Australia, Nedlands, Australia; 2 Perron Institute for Neurological and Translational Science, Perth, Western Australia Australia; 3 Medical School, University of Notre Dame, Fremantle, Western Australia Australia; 4 Department of Orthopaedics, Sir Charles Gairdner Hospital, Perth, Western Australia Australia; 5 Division of Orthopaedic Surgery, Department of Surgery, Guangdong General Hospital, Guangdong Academy of Medical Science, Guangzhou, Guangdong China

**Keywords:** Tendon-derived stem cell, Tendon homeostasis, Mechanical loading, Mechanobiology, Bioreactor

## Abstract

**Background:**

Botulinum toxin (Botox) injection is in widespread clinical use for the treatment of muscle spasms and tendinopathy but the mechanism of action is poorly understood.

**Hypothesis:**

We hypothesised that the reduction of patellar-tendon mechanical-loading following intra-muscular injection of Botox results in tendon atrophy that is at least in part mediated by the induction of senescence of tendon-derived stem cells (TDSCs).

**Study design:**

Controlled laboratory study

**Methods:**

A total of 36 mice were randomly divided into 2 groups (18 Botox-injected and 18 vehicle-only control). Mice were injected into the right *vastus lateralis* of quadriceps muscles either with Botox (to induce mechanical stress deprivation of the patellar tendon) or with normal saline as a control. At 2 weeks post-injection, animals were euthanized prior to tissues being harvested for either evaluation of tendon morphology or in vitro studies. TDSCs were isolated by cell-sorting prior to determination of viability, differentiation capacity or the presence of senescence markers, as well as assessing their response to mechanical loading in a bioreactor. Finally, to examine the mechanism of tendon atrophy in vitro, the PTEN/AKT-mediated cell senescence pathway was evaluated in TDSCs from both groups.

**Results:**

Two weeks after Botox injection, patellar tendons displayed several atrophic features including tissue volume reduction, collagen fibre misalignment and increased degradation. A colony formation assay revealed a significantly reduced number of colony forming units of TDSCs in the Botox-injected group compared to controls. Multipotent differentiation capacities of TDSCs were also diminished after Botox injection. To examine if mechanically deprived TDSC are capable of forming tendon tissue, we used an isolated bioreactor system to culture tendon constructs using TDSC. These results showed that TDSCs from the Botox-treated group failed to restore tenogenic differentiation after appropriate mechanical loading. Examination of the signalling pathway revealed that injection of Botox into quadriceps muscles causes PTEN/AKT-mediated cell senescence of TDSCs.

**Conclusion:**

Intramuscular injection of Botox interferes with tendon homeostasis by inducing tendon atrophy and senescence of TDSCs. Botox injection may have long-term adverse consequences for the treatment of tendinopathy.

**Clinical relevance:**

Intramuscular Botox injection for tendinopathy or tendon injury could result in adverse effects in human tendons and evaluation of its long-term efficacy is warranted.

## Introduction

*Botulinum* toxin (Botox) is a potent neurotoxin in widespread clinical use for the treatment of a variety of musculoskeletal disorders [23, 30]. Botox acts by blocking acetylcholine release from cholinergic nerves and endplates into the neuromuscular junction, resulting in a reduction of muscular activity [29]. Botox injections have been shown to effectively reduce muscle tone, alleviating the symptoms caused by muscular spasm and providing an alternative therapy to invasive surgery when conventional therapies have failed. One of the common uses of Botox in musculoskeletal disorders is for the symptomatic treatment of cerebral palsy. Site-specific injection of Botox reduces *tibialis posterior* and *gastrocnemius-soleus* contracture, resulting in the restoration of foot position [1, 21, 30]. In addition, Botox injection is also used for restoration of the range of head and neck motions in patients with congenital/acquired muscular torticollis who have failed in response to conventional physiotherapy [12, 27] Recently, there has been an increase in clinical use of Botox injections for the treatment of tendinopathy or tendon injuries where excessive muscle contraction may contribute to inadequate healing [13]. Several studies have reported pain relief following Botox injection after achieving sufficient suppression of muscular function in lateral epicondylitis [14, 22]. A potential mechanism of improved tendon function was reported in an animal study where Botox was injected into the supraspinatus muscle for repair of rotator cuff injury showing an improvement of collagen fibres alignment at the tendon-bone interface [7].

Tendon structure and function are closely regulated by the mechanical stimulation of passive and dynamic mechanical loading that results from daily activity [15]. Regardless of its’ specific use in muscle or tendon-related disorders, intramuscular injection of Botox causes either partial or full relaxation of the muscle and a reduced mechanical force on the associated tendons. Insufficient mechanical loading on tendon has been shown to have adverse effects on tendon morphology and causing increased stiffness, collagen turn-over and tissue atrophy [2, 24, 26].

Tendon-derived stem cells (TDSC), a population of resident tendon stem cells, are known to be responsive to mechanical signal-induced stimuli within tendon tissue [20, 25, 31, 40]. As Botox injection impairs mechanical stimulation of tendon tissue in vivo, we hypothesised that intramuscular Botox injection leads to cellular dysfunction of TDSCs. In this study, we injected Botox into the quadriceps muscles in mice, examined the patellar tendon tissue structure, isolated TDSCs and generated an ex vivo tendon construct in the bioreactor. We showed that Botox injection into quadriceps muscle induced atrophy, collagen fibre misalignment and degeneration of the patellar tendon. Botox injection of this muscle also causes irreversible senescence of TDSCs through modulation of the PTEN/AKT signalling pathway.

## Materials and methods

### Animals and Botox-induced patellar tendon unloading protocol

All animal experiments were approved by the Animal Ethics Committee, University of Western Australia. A total of 36 male C57BL/6J mice (5 weeks old) were randomly assigned into two groups: a Botox (18 mice) or vehicle-only control group (18 mice). The Botox group were injected with 3 unit/kg of Botox (10 μL per injection with a Hamilton syringe) into the right *vastus lateralis* muscle of the quadriceps group. The mice in the control group were injected with an equal volume of phosphate-buffered saline (PBS) at the same site. Two weeks after injection, the mice were sacrificed, and hind limb samples were processed for either histological analysis (6 animals in each group) or TDSC isolation (12 mice in each group).

### Histological analysis of the patellar tendons

**Fig. 1:**
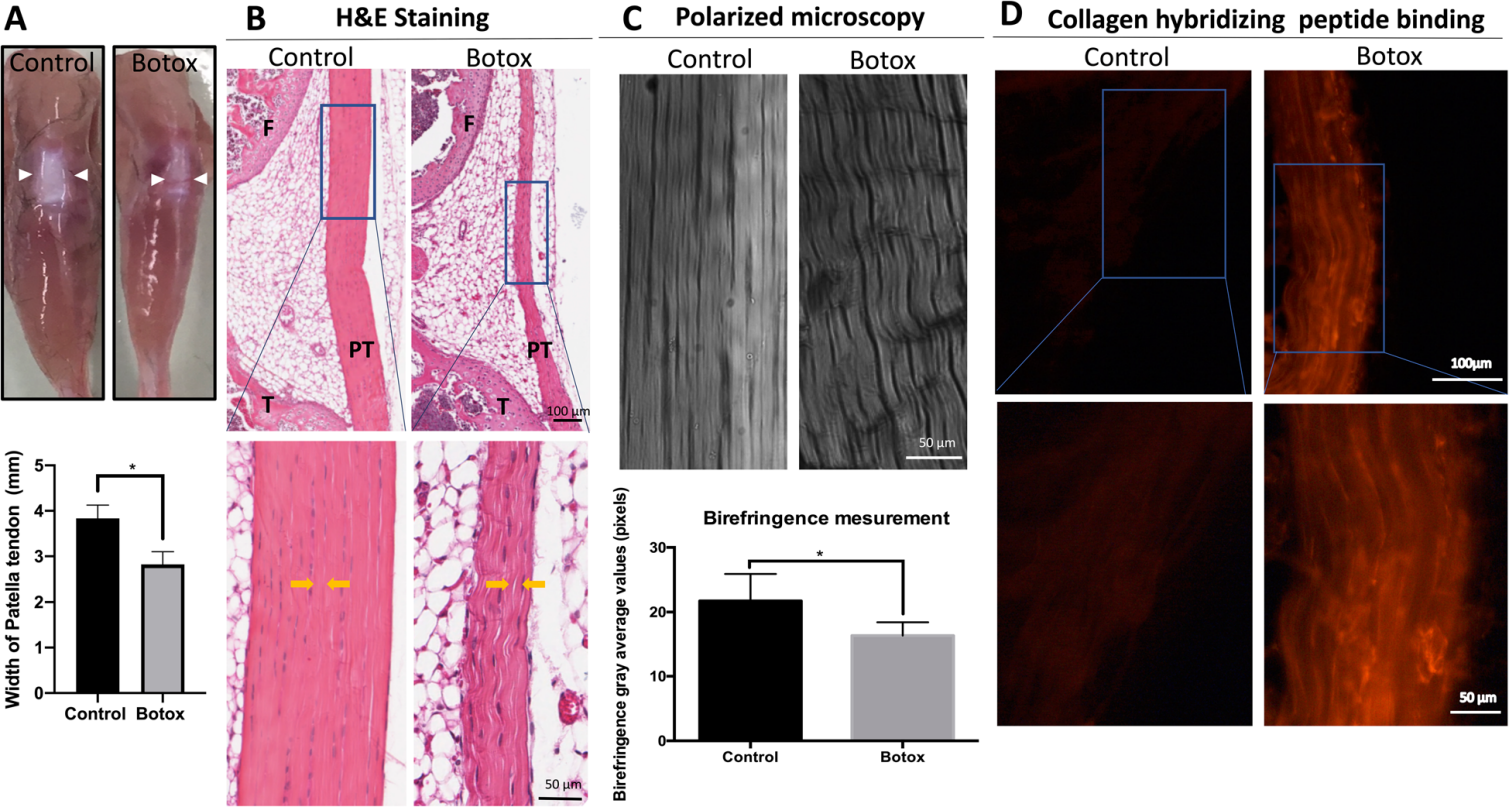
**a** Macroscopic views of the patellar tendon and surrounding musculature at 2 weeks after treatment in the contra-lateral control or Botox injected leg, and the quantitation of tendon width (*n* = 3, mean ± SD; *p < 0.05). **b** H&E staining of the patellar tendon in the control and Botox-treated group. Yellow arrows delineate the decreased width of extracellular matrix in the Botox group compared to the control group. **c** Polarised microscopic examination of the Botox and control group showing the crimps on collagen fibres in Botox group and the quantification of polarised microscopic images (*n* = 3, mean ± SD; *p < 0.05, **p < 0.005). **d** Collagen fragmentation as detected by collagen hybridising peptide shows more collagen degeneration in the Botox-injected group

Two weeks after Botox injection into the quadriceps, mice were euthanized and the hind-limbs were harvested and macroscopically observed. The maximal patellar tendon width was recorded before fixation (Fig. 1a). Then whole hind-limbs were immerse-fixed in 4% paraformaldehyde in PBS (PFA) for 48 h. The tendons were then rinsed thoroughly with PBS, dehydrated in increasing gradients of ethyl alcohol, embedded in paraffin and sectioned at 5 μm. These sections were de-waxed, rehydrated through successive decreasing gradients of ethyl alcohol, stained with haematoxylin and eosin (H&E) and mounted with coverslips using aqueous mounting medium (CV MOUNT, Leica).

### Collagen mimic peptide (CMP) staining

Immediately after harvest, hind-limb samples were frozen by immersion in liquid nitrogen. Optimal cutting temperature (OCT) compound was dropped into a plastic cryomold, samples were critically oriented and extra OCT applied until the tissue was completely covered. The cryomolds were then wrapped with aluminium foil rapidly frozen at − 80 °C and sectioned at 7 μm onto TESPA-coated slides. Excess OCT was removed by immersion in PBS before CMP staining. The unfolded triple helical collagen molecule where collagen denaturation occurs was detected with 5-FAM conjugated collagen mimic peptide (CMP; 3Helix, Inc.). The CMP solution (10 μM, in PBS) was heated to 80 °C for 5 min in a heat block and immersed in an ice-water bath for 60 s to return the solution to room temperature and prevent damage to the samples. Cooled CMP solution was added, and the samples incubated overnight at 4 °C. After staining, slides were washed in three successive changes of PBS for 5 min each at room temperature, covered with mounting medium and cover-slipped prior to observation using standard fluorescence microscope with FITC filter.

### Polarisation microscopy

After histological analysis, unstained 5-μm-thickness tissue slides were used to evaluate collagen bundle organisation using birefringence microscopy (Olympus IX8). In brief, after selecting the DIC objective and setting the condenser position to blank, samples were focused, and Kohler alignment was performed. The first and second polarising filters were inserted and adjusted to achieve the optimal polarised image. Birefringence was measured as greyscale intensity in ImageJ (Wayne Rasband, National Institute of Health, USA).

### Immunofluorescence labelling and confocal imaging

For isolated cells, TDSCs were plated on circular coverslips placed in 24-well plates at a density of 10^4^ cells/well. After attachment, cells were fixed in 4% PFA for 15 min. The fixed cells were washed with PBS 3 times and permeabilized with 0.1% Triton X-100 in PBS for 5 min at room temperature. Cells were then washed again with PBS (3 changes), and a solution of 3% BSA-PBS was applied to block non-specific antigen binding. According to the manufacturer’s instructions, primary antibodies at their recommended dilutions (in 0.2% BSA-PBS) were applied to samples and left to incubate overnight at 4 °C. The cells were washed again (3 times with PBS) for 5 min each. Secondary antibodies (diluted in 0.2% BSA-PBS as recommended) were incubated with the samples for 1 h at room temperature. After washing with PBS, staining for nuclei was conducted with Hoechst33342 (1/5000) for 15 min at room temperature. Samples were subsequently washed with PBS 3 times, mounted with anti-fade mountant medium (Diamond) and stored at − 20 °C. Confocal microscopic imaging was performed on a Nikon A1Si Microscope at a range of objective magnifications.

For examination of tendon tissues, the patellar tendons were isolated and snap-frozen in liquid nitrogen. These tendon samples were embedded in OCT compound and sectioned on a cryomicrotome into 5 μm slices. The slides containing the samples were washed with PBS to remove OCT and the same staining procedure as described above for the isolated cells was conducted. The primary antibodies used for CLSM were identical to those described for Western Blotting (above).

### Isolation and culture of TDSCs

**Fig. 2:**
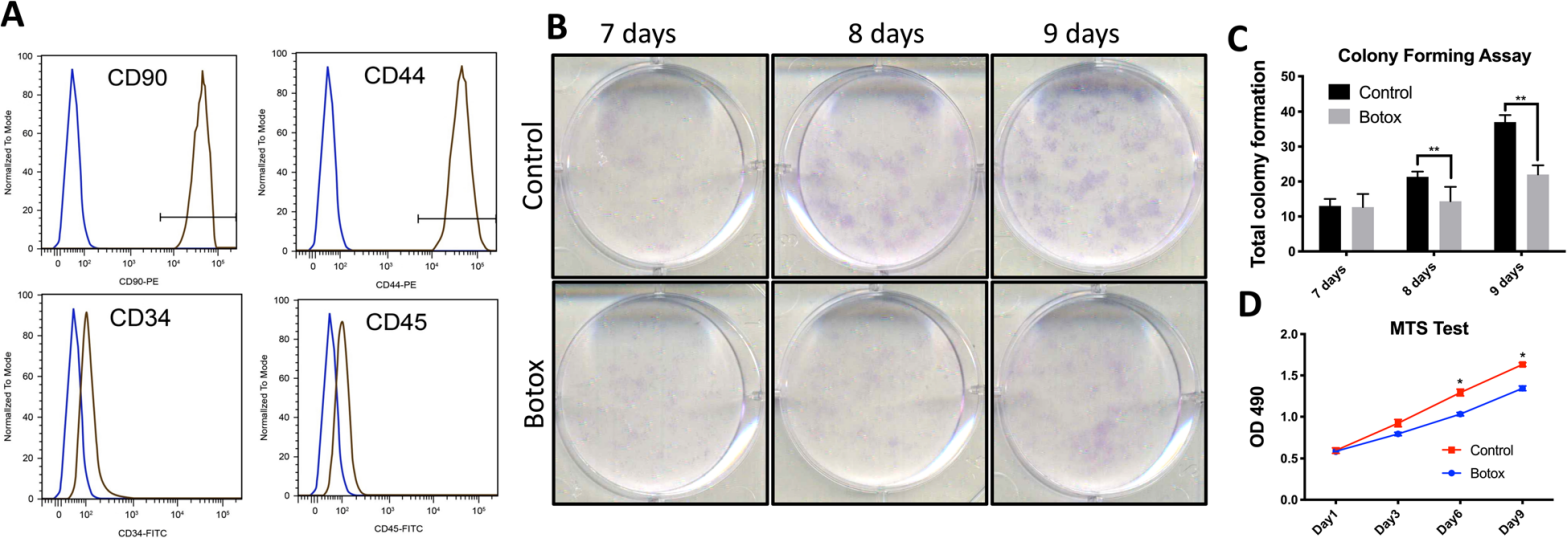
**a** Flow cytometry showing the expressions of mesenchymal stem cell markers (CD44, CD90), endothelial/haematopoietic cell marker CD34 and haematopoietic cell marker CD45. TDSCs in brown curve showed positive CD90 and CD44 but negative CD34 and CD45. **b** Colony formation assay in both control and Botox groups for 7, 8 and 9 days. **c** Quantitation of colony formation at 7, 8 and 9 days. **d** MTS test for cell viability from days 1–6 after seeding. (*n* = 3; mean ± SD; *p < 0.05, **p < 0.005)

The middle third of the patellar tendon was excised by careful dissection and subsequently rinsed in PBS containing 1% penicillin/streptomycin (P/S, Gibco™). Digestion of the patellar tendon fibres was conducted by immersion in a 3 mg/mL solution of collagenase type II (Gibco™, ThermoFisher) for 30 min. After filtration by cell strainer, residue fibres were removed. Following centrifugation, the isolated cells were cultured in a mixture containing Minimal Essential Medium (MEM Alpha, Gibco™), 10% foetal bovine serum (FBS, Gibco™) and 1% penicillin/streptomycin. Incubation conditions were 37 °C in a humidified atmosphere containing 5% CO_2_. Fluorescence-activated cell sorting was conducted to identify TDSCs (Fig. 2a) before following experiments (positive for CD90 and CD44, and negative for CD34 and CD45) [39]. Early passage (P2) were used for all experiments described below.

### Colony formation assay

To assess colony formation, TDSCs from either the control group or Botox-injected group were seeded into 6-well plates (2000 cells/well) and cultured for 7, 8 or 9 days at 37 °C in 5% CO_2_. The culture medium was then removed, and the cells were gently washed with PBS. 4% PFA solution was applied in each well for 15 min to ensure cell fixation. After washing with PBS, 0.01% (w/v) crystal violet solution (Sigma) was applied for 45 min to stain the colonies. Unbound crystal violet was removed via washing with distilled water and quantification was performed by calculating and recording the total numbers of colonies on the plate (excluding those with a diameter < 2 mm).

### Cell viability analysis

TDSCs from the control or Botox-injected group were seeded in 48-well plates (4 × 10^3^ cells/well) and at 1, 3, 6 and 9 days following seeding, an MTS assay (CellTiter®96 AQueous Non-Radioactive Cell Proliferation Assay kit; Promega, USA) was performed according to the manufacturer’s instructions. In brief, [3-(4,5-dimethylthiazol-2-yl)-5-(3-carboxymethoxyphenyl)-2-(4-sulfophenyl)-2H-tetrazolium (MTS)] was added to the medium at selected time points and the cells were incubated in dark for 3 h at 37 °C, 5% CO_2_. Optical density (OD_490_) was measured in a 96-well plate reader (Bio-Rad, Model 680, USA).

### Differentiation capacity of TDSCs

#### Osteogenesis assay

A total of 2 × 10^5^ TDSCs derived from either the control or Botox-injected mice were seeded into a 6-well plate, cultured to 80% confluence and subsequently incubated for 20 days at 37 °C, 5% CO_2_ in either basal medium (low-glucose Dulbecco’s Eagle’s medium, 10% FBS, 1% P/S) or osteogenic differentiation medium (supplemented with 20 nM β-glycerophosphate, 50 nM ascorbic acid and 1 nM dexamethasone (Sigma)). In this study, evaluation of the formation of calcium nodules reduced in tissue and cell culture was detected via a chelation process by the anthraquinone derivative Alizarin Red. For quantification, 10% w/v cetylpyridinium chloride (in 10 nM sodium phosphate, pH 7.0) was used as de-staining solution to dissolve the calcium-chelated Alizarin Red. After incubation with cetylpyridinium, the de-staining solution was harvested and read at OD_595_ in a spectrophotometric plate reader.

#### Chondrogenesis assay

To assess the ability of TDSC’s to differentiate into cartilage, 1.5 × 10^6^ cells from P2 were centrifuged at 1000 rpm for 10 min in a 15-mL polypropylene tube and subsequently cultured in chondrogenic medium consisting of high-glucose Dulbecco’s Eagle’s medium supplemented with 10 ng/mL TGF-β_3_ (R&D STSTEMS®), 500 ng/mL BMP-2 (R&D STSTEMS®), 100 nM dexamethasone (Sigma), 50 μg/mL ascorbic acid (Sigma), 40 μg/mL L-proline (Sigma) and 1:100 diluted ITS + Premix (Becton Dickinson) at 37 °C and 5% CO_2_. Following 25 days of incubation, cell pellets were gently washed with PBS and their size measured. After fixation with 4% PFA and subsequent dehydration, the cell pellets were embedded in paraffin to undergo histological evaluation (with either H&E or Safranin-O: see above).

#### Adipogenesis assay

In order to assess adipogenic differentiation, a total of 1.5 × 10^5^ cells were plated in each compartment of a 6-well plate, cultured to 90% confluence and incubated for 25 days at 37 °C, 5% CO_2_ in basal medium (high-glucose Dulbecco’s Eagle’s medium, 10% FBS, 1% P/S) and adipogenic differentiation medium (basal medium supplemented with 2 mg/mL insulin (Sigma), 10 mg/mL 3-Isobutyl-1-methylxanthine (IBMX, Sigma), 50 μM indomethacin (Sigma), 500 nM dexamethasone (Sigma)). Cells were subsequently washed with PBS and fixed with 4% PFA. Oil Red staining was performed by adding 2 mL of Oil Red staining solution at a concentration of 0.05% (w/v in isopropanol) and subsequent incubation for 15 min. After washing with 60% isopropanol, the plates were placed for air dry for 30 min and observed under a microscope.

#### Tenogenic differentiation assay

Passage 2 TDSCs were cultured to 100% confluence in a T75 flask then stimulated to differentiate into tenocytes by culturing for 7 days with complete medium supplemented with 25 ng/mL connective tissue growth factor (CTGF; PeproTech, Rocky Hill, NJ, USA) and 4.4 ng/mL ascorbic acid to promote extracellular matrix production [39]. Monolayer cell sheets were detached with 0.25% trypsin and attached to the hook at the length of 10 mm to build a tendon construct. A gross view of each construct was photographically recorded after 10 days of culture. In the uniaxial mechanical loading model, a 10-mm length tendon construct was assembled in the bioreactor and the culture conditions were developed and optimised as previously described [36]. In brief, the tendon construct was cultured in a bioreactor with optimised loading regime (6% strain, 0.25 Hz, 8 h/day followed by 16 h rest) for 5 days at 37 °C, 5% CO_2_. At the conclusion of the experiment, tendon construct samples were fixed in 4%PFA and H&E histological staining was performed as described above.

### Quantitative PCR (qPCR)

The qPCR protocol was performed as previously described [4]. In brief, TDSC’s were seeded in a 6-well plate for 7 days and mRNA was extracted from cells using PureLink™ RNA Mini Kit (Invitrogen, ThermoFisher Scientific, USA) according to the manufacturer’s instructions. Complementary DNA (cDNA) was synthesised using GoScript™ Reverse Transcription Kit (Promega). Real-time PCR (RT-PCR) was performed using iQ™ SYBR® Green Supermix according to the manufacturer’s instructions. The RT-PCR was repeated using 3 individual samples from each group and performed in triplicate in every RT-PCR setting. The relative gene expression levels of osteogenesis, chondrogenesis, tenogenesis and cytokines (MMP-13 and TNF-α) were obtained by normalising against a housekeeping gene (36B4). 36B4 has been verified as a stable housekeeping gene in previous study [39]. Primers for the selected genes are listed in Table 1.

**Table 1:** Primers for the selected genes

Gene	Primer sequence	
	Forward 5′- > 3′	Reverse 5′- > 3′
ALP	GAAGCTCTGGGTGCAGGATAG	GGACCGTCCACTGTCACTTT
BMP2	CCCCAAGACACAGTTCCCTA	GAGACCGCAGTCCGTCTAAG
RUNX2	GCCGGGAATGATGAGAACTA	GGACCGTCCACTGTCACTTT
Aggrecan	AAGGACGAGTTCCCTGGAGT	CTGGGGATGTCGCATAAAAG
SOX9	AGCTCACCAGACCCTGAGAA	TCCCAGCAATCGTTACCTTC
Scleraxis	CCCAAACAGATCTGCACCTT	GGCTCTCCGTGACTCTTCGA
Tenomodulin	AGAATGAGCAATGGGTGGTC	CTCGACCTCCTTGGTAGCAG
MKX	CTGGACAATCCACACACAGG	TCTTCGTAGGGTACGGGTTG
36B4	CTTCCCACTTGCTGAAAAGG	CGAAGAGACCGAATCCCATA

### Western blot

A total of 5 × 10^5^ cells were seeded in each well of a 6 well plate prior to washing with PBS 3 times to remove the culture medium, followed by incubation with 300 μL of complete lysis buffer (RIPA Buffer supplemented with 100 μg/mL phenylmethanesulfonyl, 1x proteinase inhibitor, 1 mM sodium orthovanadate, 500 μg/mL DNase) for 15 min. Cellular debris was pelleted, and supernatants were harvested into fresh 1 mL tubes. After quantitation with Bio-Rad Protein Assay (Bio-Rad), samples were stored at − 20 °C. For protein separation, 1.5 mm 10% SDS-PAGE stacking and running gels were set up and 20 mg boiled protein from each sample with 1x protein loading dye was loaded into the gel. After separation, proteins were transferred to H-bone membrane. After transferring, blocking step using 5% skim milk was performed for 1 h at room temperature. After washing with TBS-Tween, the primary antibody in 1% skim milk was incubated overnight at 4 °C. Samples were washed for 5 min 3 times to remove the residual primary antibody and the secondary antibody was added and incubated for 1 h. Membranes were washed with TBS-Tween (5 min, 2 times) followed by TBS (5 min, 2 times). Chemiluminescence from Western Lightning® (PerkinElmer, Inc., USA) was applied and incubated with the membrane for 2 min. Detection was performed using ChemiDoc™ MP Imaging System (Bio-Rad Laboratories, Inc., USA).

All the primary antibodies used for Western Blotting were purchased from either Abcam® (anti-CDKN2A/p19ARF ab80, anti-p16 ARC ab51243, anti-Tenomodulin ab2023276 and anti-MMP13 ab39012, anti-MMP9 ab38898, anti-beta Actin SP124) or Cell Signalling Technology® (anti-p53 1C12,anti-AKT (pan) C67E7, anti-P-PETN 9551P, anti-P-AKT (T308) 5106S, anti-P-AKT (S473) 4051S).

### Statistical analysis

Before conducting this study, we performed a power calculation based in our preliminary findings. The sample calculation was based on the MTS results between 2 groups at 9 days. A two-tail Student’s *t* test of 0.80 power and a 0.05 level of significance were achieved with 3 specimens per group.

Each experimental group had 3 internal replicates and was performed at least 3 times. A 2-tailed Student’s *t* test and 1-way ANOVA, followed by Tukey’s post hoc test (GraphPad 5.0; GraphPad Software, La Jolla, CA, USA), were used for determining the statistical significance (*p* < 0.05) in the 2-group and multi-group comparisons, respectively.

## Results

### Injection of Botox into quadriceps muscle causes patellar tendon atrophy

A single injection of Botox into the right *vastus lateralis* muscle of the quadriceps in mice caused loss of muscle contractility and reduction in loading on the patellar tendon. After a period of 2 weeks, macroscopic examination of the patellar tendon revealed that the dimensions of patellar tendons in mice injected with Botox were reduced (particularly in the proximal third) compared to those of the vehicle-only control group (Fig. 1a). H&E staining showed that patellar tendon morphology in control mice had regularly spaced collagen bundles that were well aligned with tenocytes (Fig. 1b). In contrast, patellar tendons in the Botox-injected group display atrophic features, including reductions in tissue volume and extracellular matrix (Fig. 1b and delineated by the yellow arrow) as well as collagen fibre crimping and misalignment. Polarised microscopic examination confirmed that patellar tendons from the control group displayed compacted and well-orientated collagen bundles compared to those in the Botox group (Fig. 1c). To assess whether disorientated collagen bundles in the Botox-treated group were due to degradation of collagen fibres, we employed the CMP binding assay which detects denatured collagens in tendon [10, 11]. Our results showed that atrophic tendons in the Botox-treated group have increased CMP binding of the conjugated fluorescent element along the collagen fibres as compared to that in the control group (Fig. 1d). Collectively, these results demonstrate that intramuscular Botox injection causes tendon atrophy and degradation of the collagen matrix.

### Intramuscular Botox injection impairs the growth and differentiation potential of TDSCs

We next hypothesised that tendon atrophy after intramuscular Botox injection is mediated via an indirect effect of the decreased muscle activity on tendon cell turnover and differentiation. Thus, we isolated TDSCs from patellar tendons of Botox-treated or vehicle-only control mice and evaluated their respective viabilities and capacities for differentiation. The colony formation assay revealed that the numbers of TDSCs colony forming units in the *Botox*-injected group were significantly reduced in comparison to vehicle-only controls (Fig. 2b, c). Calculation of cell viability using the MTS assay revealed a significant reduction in cell numbers in the Botox group at days 6 and 9 after seeding compared to the control (*n* = 3, *p* < 0.05) (Fig. 2d). Together, these results showed that injection of Botox into quadriceps muscle impairs cell growth of TDSCs.

**Fig. 3:**
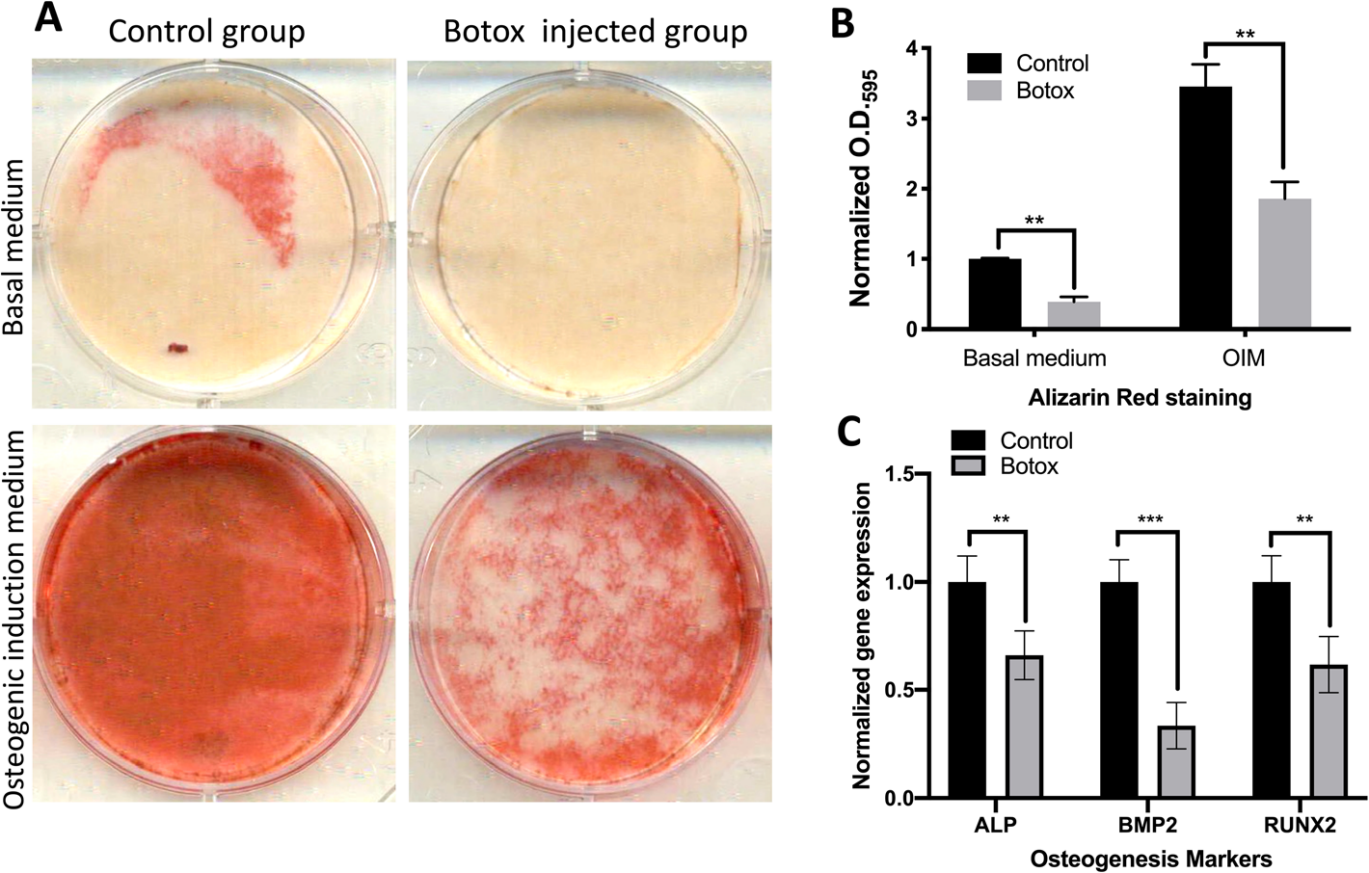
**a** Alizarin Red staining for calcium nodule formation in the control and Botox-injected group cultured in either basal or osteogenic induction medium (OIM) showing the decreased calcium nodules formation in the Botox group. **b** Quantitation of Alizarin Red by read at 595 nm of destaining solution by 96-well plate reader and normalised to basal-medium control group (*n* = 3; mean ± SD; *p < 0.05, **p < 0.005, *** *p* < 0.001). **c** mRNA expressions of osteogenic differentiation markers (ALP, BMP2 and RUNX2) in the control and Botox group. Individual mRNA expression levels were first normalised to internal control (36B4) and then normalised against mRNA expression level from the control group. (*n* = 3; mean ± SD; **p < 0.005, ***p < 0.001)

**Fig. 4:**
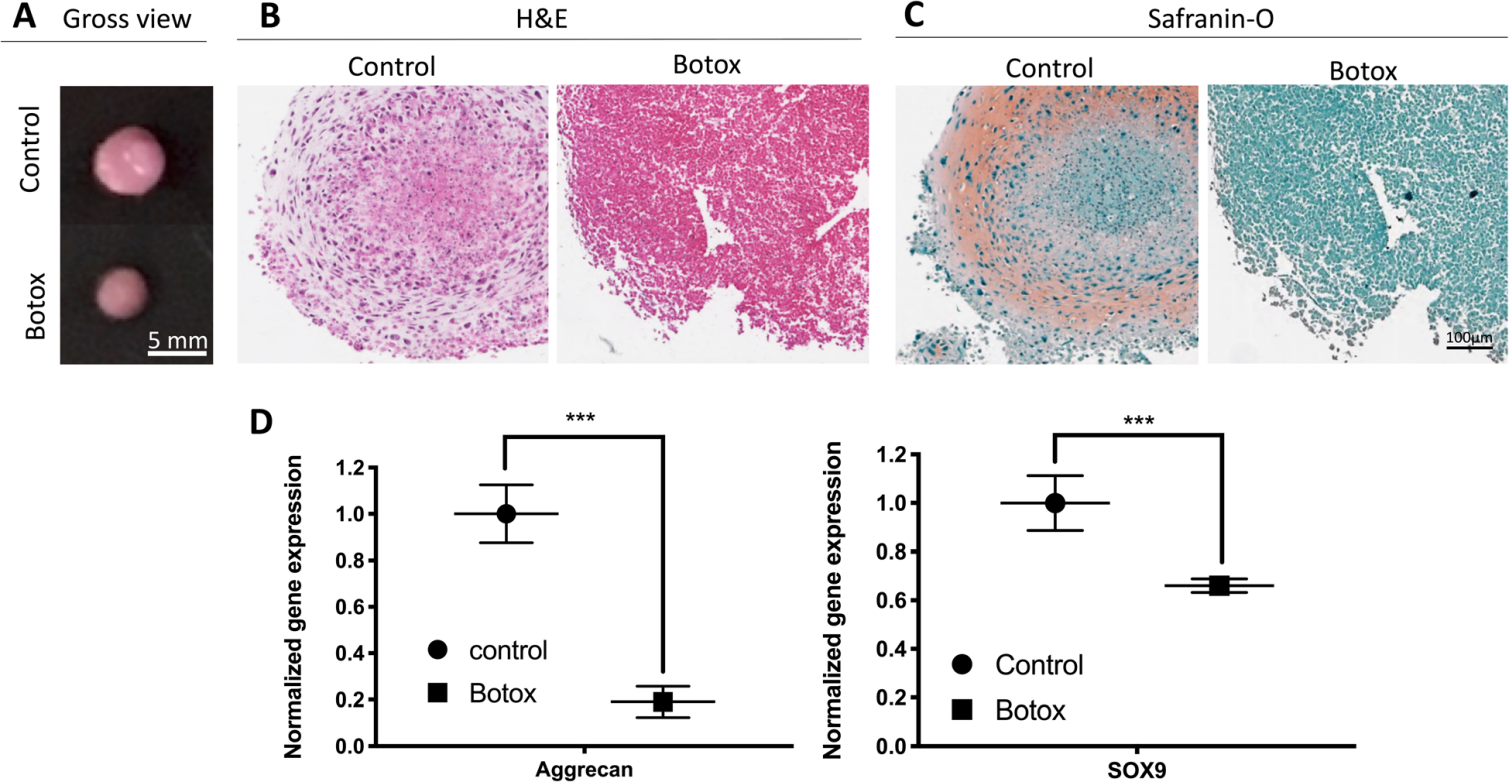
**a** Gross observation of pellet culture for chondrogenesis of TDSCs in the Botox and control group showing the larger size of the pellet culture system from control. **b** H&E staining of pellet culture in Botox and control group showed better cell population in the control compared to the Botox group. **c** Safranin-O staining of the pellet culture system demonstrated greater proteoglycan deposition in control. **d** mRNA expression of chondrogenic differentiation markers (Aggrecan and SOX9) in the control and Botox group. Individual mRNA expression levels were first normalised to internal control (36B4) and then normalised against mRNA expression level from the control group (*n* = 3; mean ± SD; ***p < 0.001)

**Fig. 5:**
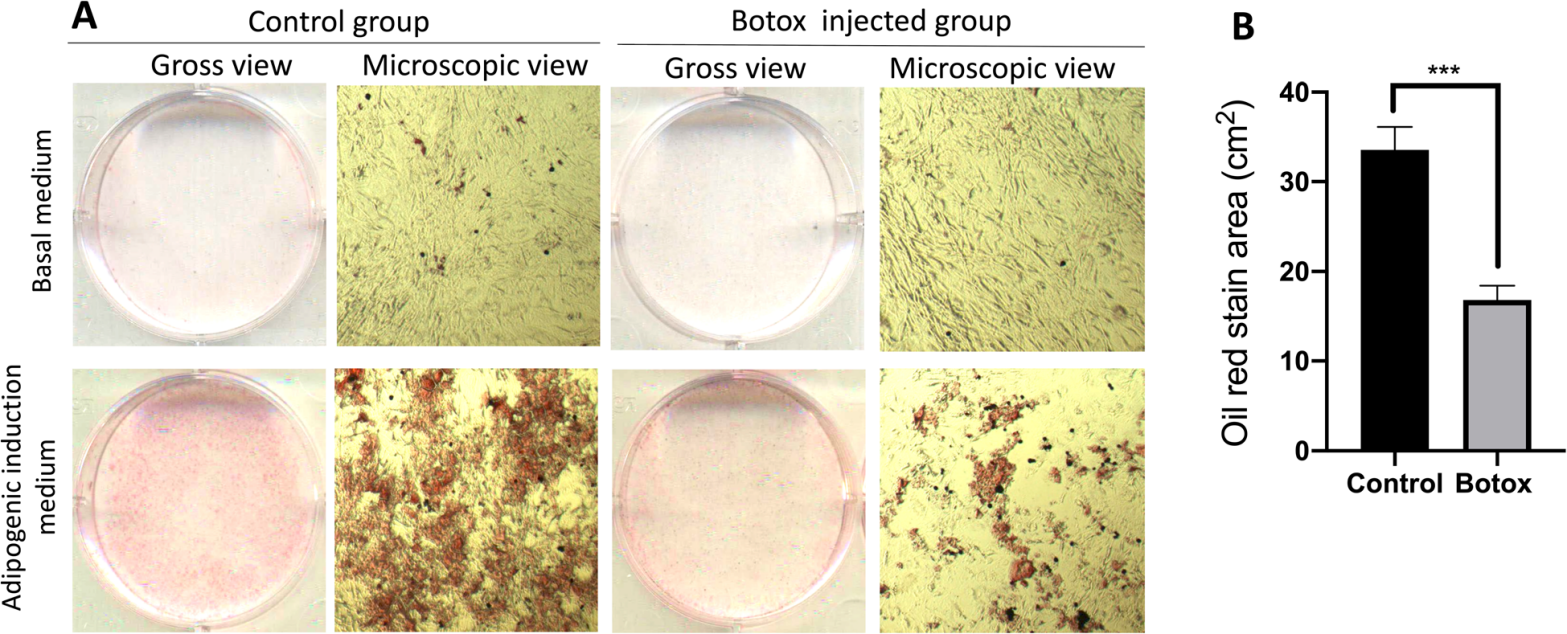
**a** Examination of adipogenesis via Oil-Red staining for lipid formation in the Botox group and control group. **b** Quantitation of Oil Red staining by measurement of stain area (*n* = 3; mean ± SD; ***p < 0.001)

TDSCs are multipotent progenitor cells capable of differentiating into tendon, cartilage, bone and adipose tissue [17, 39]. Therefore, we determined if injection of Botox into quadriceps muscle affects the multi-lineage differentiation potential of TDSC. The TDSCs from the Botox-treated group produced fewer calcium nodules in osteogenic differentiation medium (*p* < 0.05; Fig. 3a, b). There was also a reduction in gene expression for osteogenic markers (40% reduction in ALP, 60% reduction in BMP2 and 47% reduction in RUNX2; *p* < 0.05; Fig. 3c) in the TDSCs from the Botox-treated group. Measurement of chondral pellet formation by TDSC revealed that TDSCs from the Botox-treated group have a reduced size compared to the control group (Fig. 4a). Morphological observation of sections from embedded pellets showed fewer chondrocyte-like cells and less regular extracellular matrix in the Botox injection group than in the control group (Fig. 4b). Safranin-O staining showed a reduction in proteoglycan deposition in pellets from the Botox-treated group (Fig. 4c). In addition, the mRNA expression for chondrogenic-specific markers (Aggrecan and SOX9) was decreased in TDSCs from the Botox-treated group (80% reduction in Aggrecan and 35% reduction in SOX9, *p* < 0.001; Fig. 4d). Oil-Red staining indicated a significant reduction in lipid drop formation indicating a diminished adipogenic capacity of TDSCs from the Botox-treated group (Fig. 5). These results support the concept that injection of Botox into quadriceps muscle impairs proliferation and multipotent differentiation capacity of patellar tendon TDSCs.

### TDSCs from Botox-treated mice grown in an ex vivo bioreactor show a reduced capacity for extracellular matrix formation and tenogenesis

**Fig. 6:**
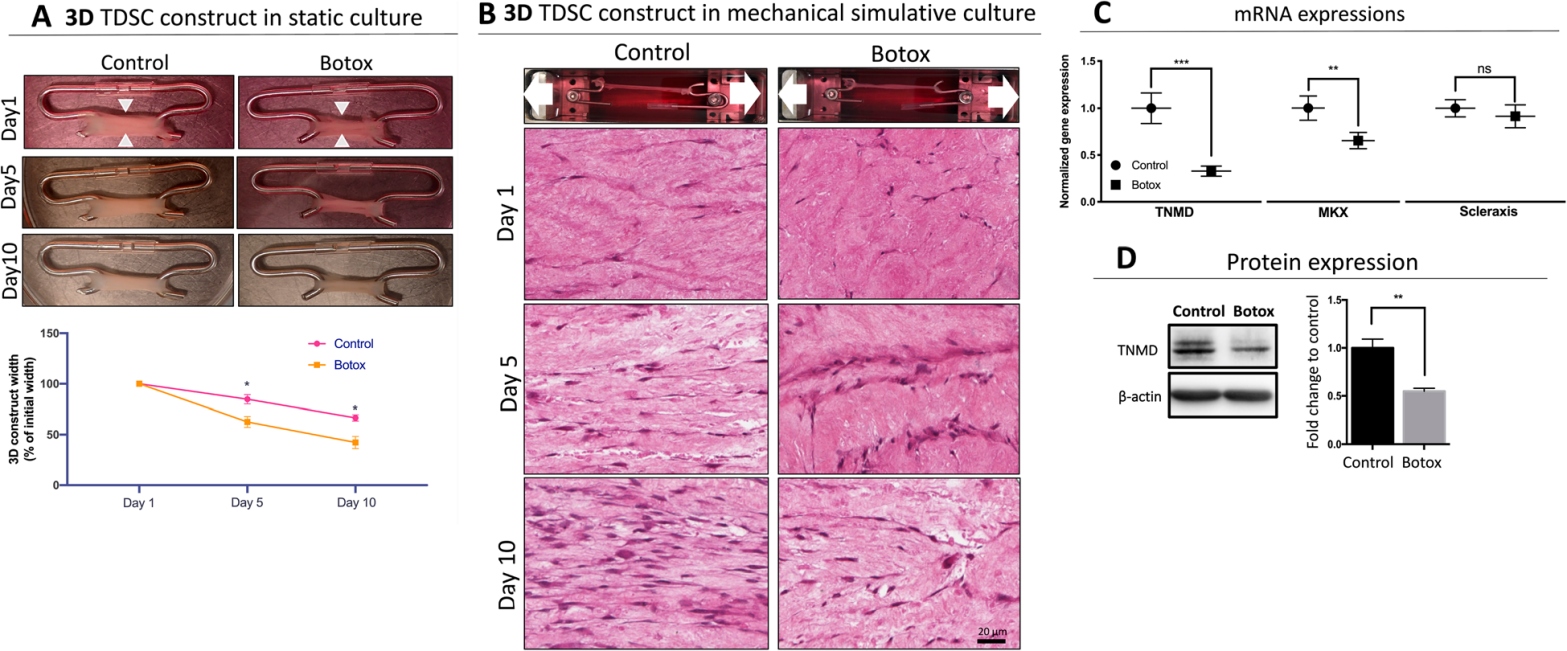
**a** Gross view of in vitro static culture of tendon construct using isolated TDSC from the control and Botox group, and the contraction rate was measured by the width of the construct (indicated by white arrowhead). The width of Botox showed faster contraction rate (*n* = 3; mean ± SD; *p < 0.05, **p < 0.005). **b** Schematics of uniaxial mechanical loading system and H&E staining of post-loading tendon construct. The direction of uniaxial mechanical stretching is indicated by the white arrows. **c** mRNA expression of tendon specific markers (TMND, MKX and Scleraxis) from both the Botox and control groups. Individual mRNA expression levels were first normalised to internal control (36B4) and then compared to mRNA expression level from the control group (*n* = 3; mean ± SD; **p < 0.005, ***p < 0.001). **d** Western blot of TMND in both group and quantification and were normalised to beta-actin. Results were quantified by greyscale density (*n* = 3; mean ± SD; **p < 0.005, ***p < 0.001)

Immobilisation of muscle following injection of Botox is reversible after the first few weeks [8, 33]; therefore, we posit that the mechanically induced reduction of growth and differentiation potential of TDSCs is also reversible and tendon atrophy can recover in vivo. However, it is not clear that if the population of TDSC that has suffered from mechanical deprivation is capable of forming tendon tissue. To test this, we used an isolated bioreactor system to culture tendon constructs to determine if TDSCs from the Botox-treated group can restore normal function by forming neo-tendon tissue and expression of tendon markers (Tenomodulin, MKX and Scleraxis) after induction of mechanical loading. TDSCs isolated from Botox injected animals experiencing mechanical deprivation in a static culture results in a reduction of neo-tendon tissue formation compared to the vehicle-only control-treated group. Measurement of tendon constructs revealed a significant reduction in the widths between TDSCs obtained from either control or Botox-treated groups in static culture (30% in control vs 55% in Botox; *p* < 0.05; Fig. 6a). These findings are consistent with histological observations of in vivo atrophic patellar tendons from the Botox-treated group (Fig. 1b). In a previous study, mechanical loading can improve recovery following tendon atrophy [38]. However, TDSCs grown in the bioreactor from the Botox-treated group failed to restore tenogenic capacity. Specifically, TDSCs from the vehicle-only control group demonstrated neo tendon tissue formation with a high cellular density, more organised extracellular matrix structure and collagen alignment whereas the cell population in the Botox-treated group was reduced with an associated disruption of extracellular matrix morphology and no obvious neo-tendon formation (Fig. 6b). Examination of tenogenic-specific markers revealed that mRNA levels of TNMD and MKX were reduced in the Botox-treated group (60% reduction in TNMD and 50% reduction in MKX; *p* < 0.01, Fig. 6c and d, respectively), but there was no significant change in the mRNA levels of Scleraxis between the two groups. Together, these results demonstrate that TDSCs from Botox-treated mice fail to restore tenogenic differentiation after appropriate mechanical loading. It appears that the reduced viability and diminished differentiation of TDSCs after injection of Botox into quadriceps muscle are irreversible in our bioreactor experiments.

### Botox-treatment causes PTEN/AKT-mediated cell senescence of TDSCs

**Fig. 7:**
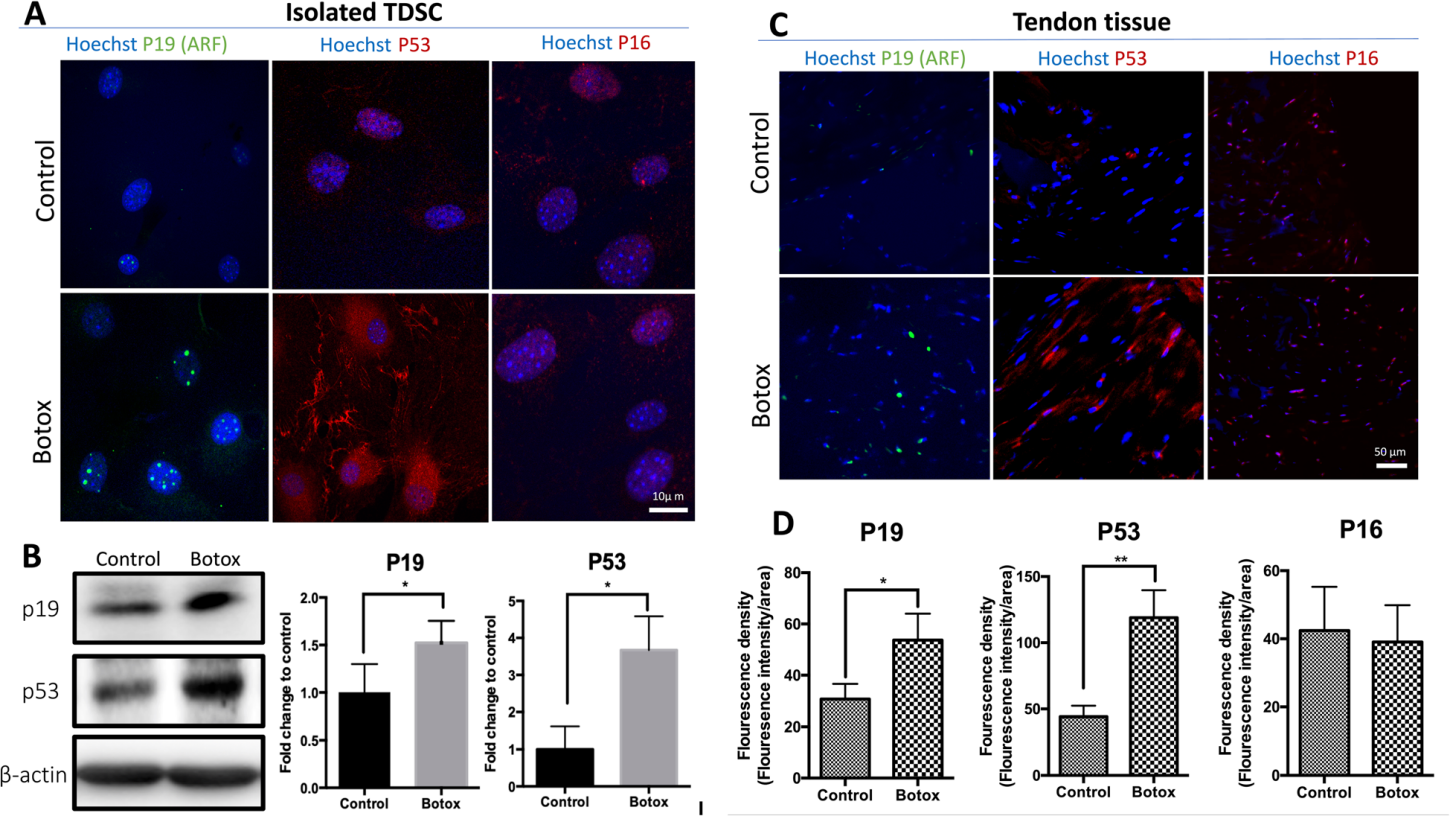
**a** Immunofluorescent staining of senescent markers (P19, P53 and P16) of isolated TDSCs from control and Botox tendon showed increased accumulation of P19 and P53 in the Botox group. But P16 was not significantly changed between both groups. **b** Western blot of protein expression of P19 and P53 from isolated TDSCs were normalised to beta-actin and showed increased translational level of P19 and P53 in the Botox group. Results were quantified by greyscale density (*n* = 3; mean ± SD; *p < 0.05, **p < 0.005). **c** Immunofluorescence staining of P19, P53 and P16 on tendon tissue exhibited higher production of P19 and P53 in the Botox group and again not P16. **d** Quantification of fluorescence density of P19, P53 and P16 in tendon tissue. Blinded analysis was performed for quantification (*n* = 3; mean ± SD; *p < 0.05, **p < 0.005)

**Fig. 8:**
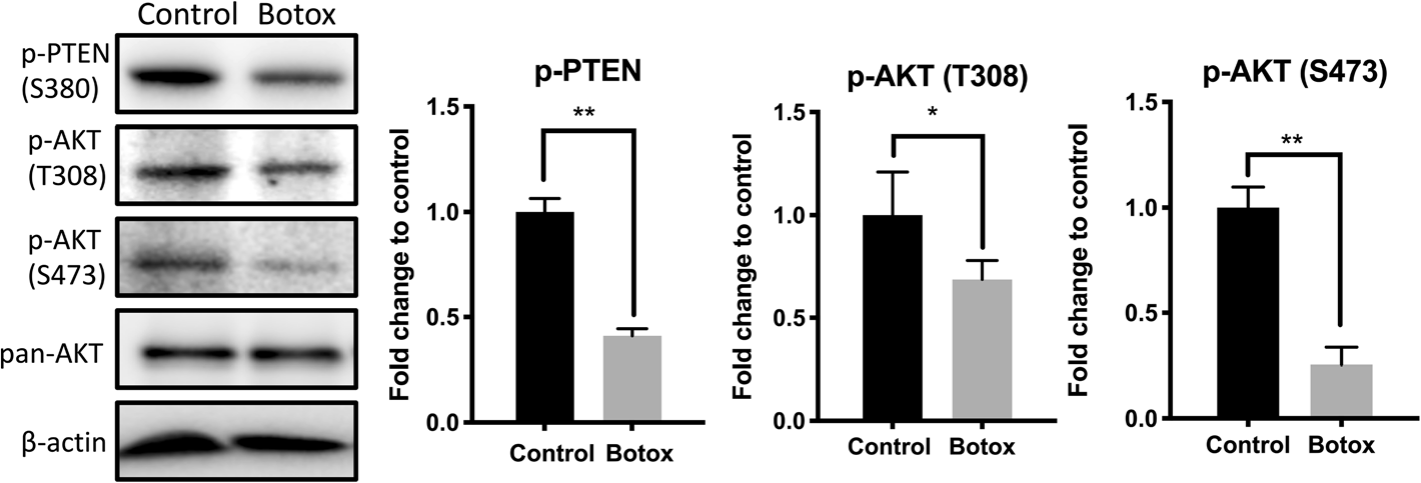
Western blot of p-PTEN, p-AKT (T308), p-AKT (S473) and pan-AKT in the control and Botox group were normalised to beta-actin and showed the decreased phosphorylation of both PTEN and Akt (on both T308 and S473 site), and the results were quantified by greyscale density (*n* = 3; mean ± SD; *p < 0.05, **p < 0.005)

To determine why re-introduction of mechanical loading in Botox-treated TDSCs fails to generate neo-tendon-like tissues in 3D bioreactor culture, we hypothesised that mechanical deprivation as a result of paralysed nerve function causes senescence of TDSCs. Previously, the PTEN/Akt pathway has been shown to play a pivotal role in regulating cell proliferation and senescence [35, 43]. We thus explored the potential mechanism of PTEN/AKT axis-induced cells senescence. Accumulation of cell senescence markers of p19 and p53, but not P16 was seen in TDSCs in the Botox group (Fig. 7a). Western blot analysis further confirmed a higher level of p19 and p53 protein expression in TDSCs in the Botox group than that in the control group (Fig. 7b). On tissue level, CLSM images analysis of tendon tissue also showed a greater level of p19 and p53 production in the Botox group as compared to the control (*n* = 3, *p* < 0.05) (Fig. 7c, d). Further examination of PTEN/AKT signalling pathway by Western blots revealed decreased phosphorylation on PTEN and AKT at both sites (T308 and S473) in TDSCs in the Botox group (Fig. 8). Together these results suggested that injection of Botox into quadriceps muscle causes PTEN/AKT-mediated cell senescence of TDSCs.

## Discussion

Tendon senses mechanical loading from muscle [6]. Previous studies have shown that the tendon only received a “sweet spot” of proper mechanical loading that can induce tendon anabolic effect [36, 37]. At 6% cyclic tensile loading of the “sweet spot” mechanical loading, tendon showed well-aligned collagen fibre and cell function and maintain the anabolic status [37]. However, when tendon experiencing over loading at 9% cyclic tensile loading, it displays rupture of collagen fibres. On the other hand, when tendon subjected to insufficient mechanical loading of 3% cyclic tensile loading, tendon tissue displays matrix degeneration and increased production of MMP-1, 3 and 12. In further, when there is non-mechanical loading (mechanical loading deprivation), tendon tissue exhibited disorganised collagen fibre and increased cell apoptosis [37].

**Fig. 9:**
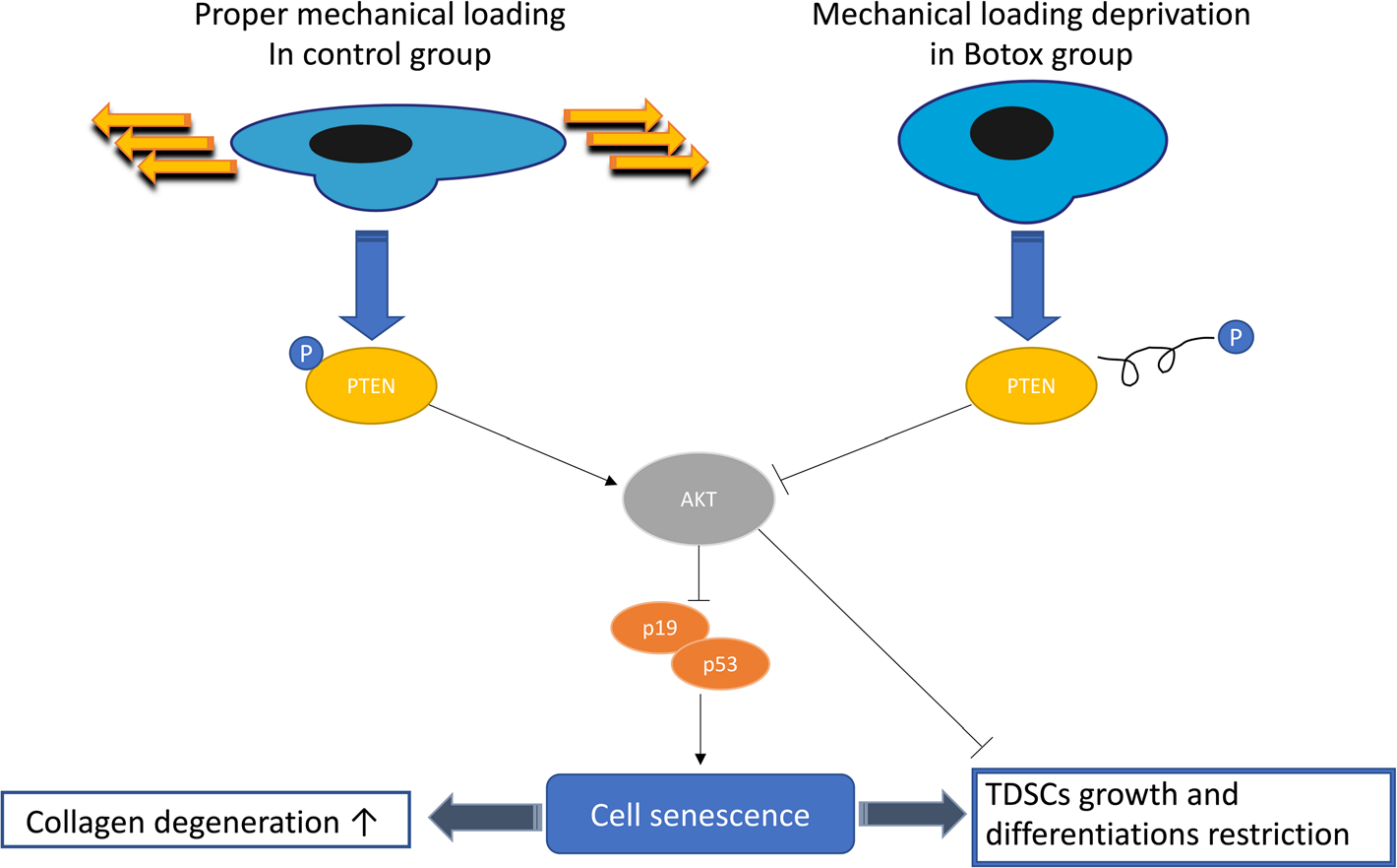
The schematic of how mechanical loading deprivation affect TDSCs through alteration of PTEN/AKT pathway and result in cell senescence

In this study, we showed that intramuscular Botox injection cause deprivation of mechanical loading of tendon, resulting in the induction of tendon atrophy, disorientation and degeneration of collagen fibres [6]. We also found that cell proliferation and potentiality of TDSCs was impaired under Botox-induced loading deprivation. Also, senescence phenotype of TDSCs evidenced by increased levels of senescence markers p19/p53 was observed in the Botox group. Further investigations revealed the dysfunction of PTEN/AKT due to the increased activation of PTEN and subsequent decreased AKT activation which is the possible mechanism of compromised TDSCs function (Fig. 9).

TDSCs are a cell population with stem cell properties essential for tendon tissue maintenance and repair through self-renewal and proliferation [25, 41, 42]. Dysfunction of TDSCs has been found to contribute to tendon tissue ageing due to decreased self-renewal and clonogenic deficits [16]. Some studies have shown that cyclic tensile strain in vitro results in upregulation of proliferation and tenogenic differentiation of TDSCs [9, 40]. Furthermore, TDSCs also have the potential for trilineage differentiation (osteogenesis, chondrogenesis and adipogenesis), and this capacity can be enhanced by adequate mechanical stimulation [17, 31, 34]. We showed that TDSCs isolated from the tendons with Botox injection into associated muscles display reduced proliferation, colony formation capacity and trilineage differentiation capacity (osteogenesis, chondrogenesis and adipogenesis). Additionally, tenogenesis was diminished as evidenced by decreased transcription and translation of tendon markers (TNMD and MKZ) and less tissue substance in tendon construct. Even though the tendon construct was subjected to uniaxial mechanical loading for 10 days, extracellular matrix was not realigned, suggesting that functions of TDSCs from the Botox group were not restored during this period. In our model, we also found that regulation of tenogenesis by mechanical loading is mediated through MKX [18].

Previously, PTEN has been shown to be an inhibitor of AKT activity and phosphorylation of PTEN (p-PTEN) which can lead to the inhibition of cell growth and differentiation [3, 28]. In addition, PTEN/AKT signalling pathway was involved in the regulation of cell senescence in tendon [35, 43]. Senescent cells sustain the state of cell-cycle arrest in which proliferation was impaired, and senescence has a significant effect on mesenchymal stem cells (MSCs) differentiation [5, 19]. Senescence related factor p53 is a key regulator of cell senescence in initiating cell cycle arrest [32], and p19 (p14 in humans) is known to be required for the activation of p53 [19]. Considering that TDSC senescence is associated with alteration of cell fate [42], we thus speculated that Botox-induced tendon atrophy may be due to the induction of TDSC senescence by mechanical deprivation. We showed decreased p-PTEN level in TDSCs receiving Botox injection. The decreased p-PTEN suppress AKT activity as evidenced by decreased phosphorylated in both termini on AKT. Conversely, there were increased levels of cell senescence markers p19 and p53 in isolated TDSC and tendon tissue in the Botox group. These results suggest the role of PTEN/AKT in impaired cell growth and differentiation in stress-deprived tendons either by its effect on cellular senescence.

In this study, we revealed cellular and extracellular changes in stress-deprived tendons and the possible mechanism underlying this response. However, we recognise limitations in this study, including the possibility of reversibility of the atrophic tendon tissue phenotype and TDSC senescence beyond 2 weeks post Botox injection in vivo. In future studies, we should investigate the reversibility of tendon atrophy in vivo after Botox injection. Nevertheless, by applying stretch to the tendon construct in the bioreactor, we showed that cell function cannot be reversed by re-introduction of mechanical loading. In addition, it will be important to define how single or multiple Botox injections regulate the extent and duration of TDSC replicative senescence in the unloaded tendon and further elucidate the mechanism underlying this phenomenon. In addressing these limitations in further research, there is great potential for discovering preventative measures and optimising therapeutic strategies used in the treatment and surgical repair of tendon injuries.

## Conclusions

In this study, we investigated the pathological changes of the mechanically deprived patellar tendon by Botox injection at the histological and cellular level. Tendon atrophy and collagen fibre disorientation were observed in the Botox injection group. TDSCs from the Botox injection group exhibited decreased proliferation, clonogenicity and trilineage differentiation (osteogenesis, chondrogenesis and adipogenesis) capacity. Tenogenesis of TDSCs from the Botox injection group was diminished, reflecting a compromised potential of TDSCs. Even though the mechanical loading was reintroduced, 3D TDSC constructs could not form tendon-like tissue. Impaired cell proliferation and differentiation indicated cell senescence in the Botox group, and our study showed elevated levels of cell senescence regulators p19/p53 in the Botox group. This suggests that intramuscular Botox injection for tendinopathy and tendon injury may cause adverse effects in tendons.

## Data Availability

The datasets used and/or analysed during the current study are available from the corresponding author on reasonable request.

## Abbreviations

TDSC: Tendon-derived stem cell
Botox: Botulinum toxin
